# The impact of 3D printed vs. 3D virtual congenital heart models on patient and family knowledge

**DOI:** 10.3389/fped.2025.1525549

**Published:** 2025-03-14

**Authors:** Luke Zerwic, Ashray Mohan, Emily Riley, Connor Byeman, Ravi Ashwath

**Affiliations:** ^1^ Carver College of Medicine, University of Iowa, Iowa City, IA, United States; ^2^ Stead Family Department of Pediatrics-Cardiology, University of Iowa, Iowa City, IA, United States; ^3^ CHRISTUS Children’s Hospital, Baylor College of Medicine, San Antonio, TX, United States

**Keywords:** congenital heart disease, patient education, 3D printing, cardiac models, digital models

## Abstract

**Introduction:**

Congenital heart defects (CHDs) often involve complex anatomical structures that can be challenging for patients and their families to understand. While physicians utilize various imaging techniques such as cardiac echocardiograms, CT scans, and MRIs to comprehend these complexities, the information is typically conveyed to patients and families through two-dimensional (2D) images and drawings. Traditional methods often fail to fully capture the intricate nature of CHDs. This study compared the effectiveness of 2D imaging with three-dimensional (3D) virtual and 3D printed models in enhancing the understanding of CHDs among patients and their families.

**Methods:**

Family members of patients with congenital heart disease, as well as patients aged 15 years or older, were recruited for the study. Participants were presented with an echocardiogram of their specific cardiac defect alongside an echocardiogram of a structurally normal heart for comparison. They were then randomly assigned to receive education using a 3D printed model or a 3D virtual model of their heart defect. Participants' knowledge of normal cardiac anatomy and the anatomy of their specific cardiac defect was assessed after viewing the echocardiogram (2D image) and again after reviewing the 3D models.

**Results:**

One-hundred-nine subjects participated in the study, comprising 79 family members (72.5%) and 30 patients (27.5%). Subjects showed significant improvement in their understanding of normal cardiac anatomy with both 3D printed and 3D virtual models compared to the 2D image (*p* = 0.022 and *p* = 0.012, respectively). Among the subjects, 70% in the 3D printed group and 84% in the 3D virtual group indicated a preference for the 3D models over the 2D image. Both the 3D printed, and 3D virtual model groups rated themselves as having an increased understanding of normal cardiac anatomy compared to the 2D images (*p* = 0.009 and *p* < 0.001, respectively).

**Discussion:**

These findings suggest that incorporating 3D models into the educational process for patients with congenital heart disease can lead to improved comprehension and greater satisfaction.

## Introduction

Congenital heart diseases (CHDs) affect nearly 1% of births each year, with 25% of these patients having a critical defect needing surgery or other interventions (1, 2). There are various types of congenital heart defects (CHDs), which can be categorized based on the location and nature of the defects. One method of categorization includes: CHDs involving only the large blood vessels: These defects occur outside the heart chambers and primarily affect the major arteries and veins, CHDs involving only the heart chambers: These defects are confined to the atria or ventricles of the heart and CHDs involving multiple structural abnormalities: These defects affect both the large blood vessels and the heart chambers, leading to complex congenital heart conditions (3).

Given the prevalence and severity of CHDs, it is crucial to effectively communicate information about the defect to patients and their families. Currently, counseling happens using two-dimensional (2D) images, such as echocardiograms or pictures. While these 2D images can quickly review the anatomy of a patient's defect, they often provide a superficial view of the condition. In contrast, recent advancements in three-dimensional (3D) models offer a more detailed perspective, allowing observers to hold, rotate the model and appreciate the complex anatomy of CHDs.

These new models can be used in clinics to educate patients and families about their complex cardiac conditions. One study found that instruction in clinics with 3D printed models led to an increase in 5 min per visit, which was not perceived as a significant hindrance, especially given the increase in satisfaction gained from the use of these models (4). Biglino and colleagues used a focus group approach to gather information from patients, parents, clinicians and nurses about the usefulness and potential limitations of 3D models. All four groups believed that the 3D models would be beneficial for CHD education. Interestingly, parents indicated that they preferred a patient specific model as opposed to a more generic lesion specific model (5).

Previous studies have shown that using 3D printed models to counsel family members about CHDs can produce results equal to or better than current 2D drawings (6, 7). Additional studies have demonstrated the utility of 3D printed models in preoperative consultations (8), educating and training physicians (9–12) and planning for surgical intervention (13–16).

However, gaps in the literature exist regarding the comparative effectiveness of 3D printed and 3D virtual models, particularly when tested with patients and family members dealing with complex CHDs. One study in 2021 by Awori and colleagues compared 3D printed and 3D virtual models, but only tested these models in medical personnel and parents, patients were not included in the study (17). Additionally, the number of parents that were included was relatively small at 20 subjects. The authors found that the parents perceived understanding with 3D printed and 3D virtual models were significantly higher than traditional 2D images, and that 3D printed models were more useful than digital ones. While this study was instrumental in comparing the differences between 3D printed and 3D virtual models, it lacked a comparison of the two models in both patient and family education.

Both 3D printed and 3D virtual models can be used to educate patients and their families about CHDs, each with its own strengths and weaknesses. 3D printed models allow for physical manipulation, providing a tactile learning experience, whereas 3D virtual models are accessible through a computer or phone screen. However, 3D printing is expensive and time-consuming, and only one person can access the model at a time. In contrast, 3D virtual models are an easy-to-access and inexpensive alternative, allowing multiple people to view and interact with the model simultaneously. Importantly, these models can be accessed by patients and family members at any time via a QR code, enabling them to educate others, such as friends and relatives.

This study evaluated the differences between 3D printed and 3D virtual models in improving patient and family's knowledge of complex CHDs. We also compared the effectiveness of 2D cardiac models and 3D models on the same measures. The most common CHDs that subjects experienced were coarctation of the aorta, tetralogy of Fallot, and transposition of the great arteries (see Table 1 for the complete list). We hypothesized that 3D heart models would significantly improve participants' knowledge of CHD anatomy and normal heart anatomy compared to traditional 2D imaging. Additionally, we hypothesized that 3D virtual models would lead to significantly better performance compared to 3D printed models in terms of educating patients and family members.

**Table 1:** Patient cardiac diagnosis and demographic characteristics of test takers.

Variables	Printed 3D model	Virtual 3D model	*p*-value*
	*n* = 34 patients	*n* = 39 patients	
Cardiac diagnosis, count (%)			
Coarctation of the aorta	11 (32)	13 (33)	
Tetralogy of Fallot	8 (24)	8 (21)	
TGA	5 (15)	5 (13)	
PFO/ASD	4 (12)	2 (5)	
TAPVR	1 (3)	3 (8)	
VSD	0 (0)	3 (8)	
Double aortic	2 (6)	1 (3)	
PDA	2 (6)	1 (3)	
Truncus arteriosus	1 (3)	2 (5)	
Fontan	0 (0)	1 (3)	
Test taker, count (%)	Printed 3D model *n* = 54 test takers	Virtual 3D model *n* = 55 test takers	0.88
Patient	11 (20)	13 (24)	
Mother	28 (52)	29 (53)	
Father	11 (20)	11 (20)	
Other	4 (7)	2 (4)	
Age, years			
Mean (SD)	35.2 (13.2)	35.0 (9.4)	0.93
Range	15–73	15–57	
Gender, female	39 (72)	38 (69)	0.72
Race/Ethnicity			0.10
White, Non-Hispanic	48 (89)	49 (91)	
White, Hispanic	2 (4)	3 (6)	
Other, Hispanic	0 (0)	2 (4)	
Other, Non-Hispanic	4 (7)	0 (0)	
Education			0.009
Some high school	6 (11)	2 (4)	
High school graduate	11 (20)	6 (11)	
Some college	12 (22)	8 (15)	
College graduate	20 (37)	30 (55)	
Post-graduate	5 (9)	9 (16)	

Comparison of demographic characteristics of test takers between the 3D printed and 3D virtual groups showed a significantly higher level of education for those in the virtual group. To account for this difference, statistical tests comparing responses between the 3D printed and 3D virtual groups controlled for education.

* *p*-values for group comparisons were from Pearson Chi-square test for nominal categorical variables, Wilcoxon-rank sum test for education, and two-sample t-test for age.

## Methods

### Model creation

**Figure 1:**
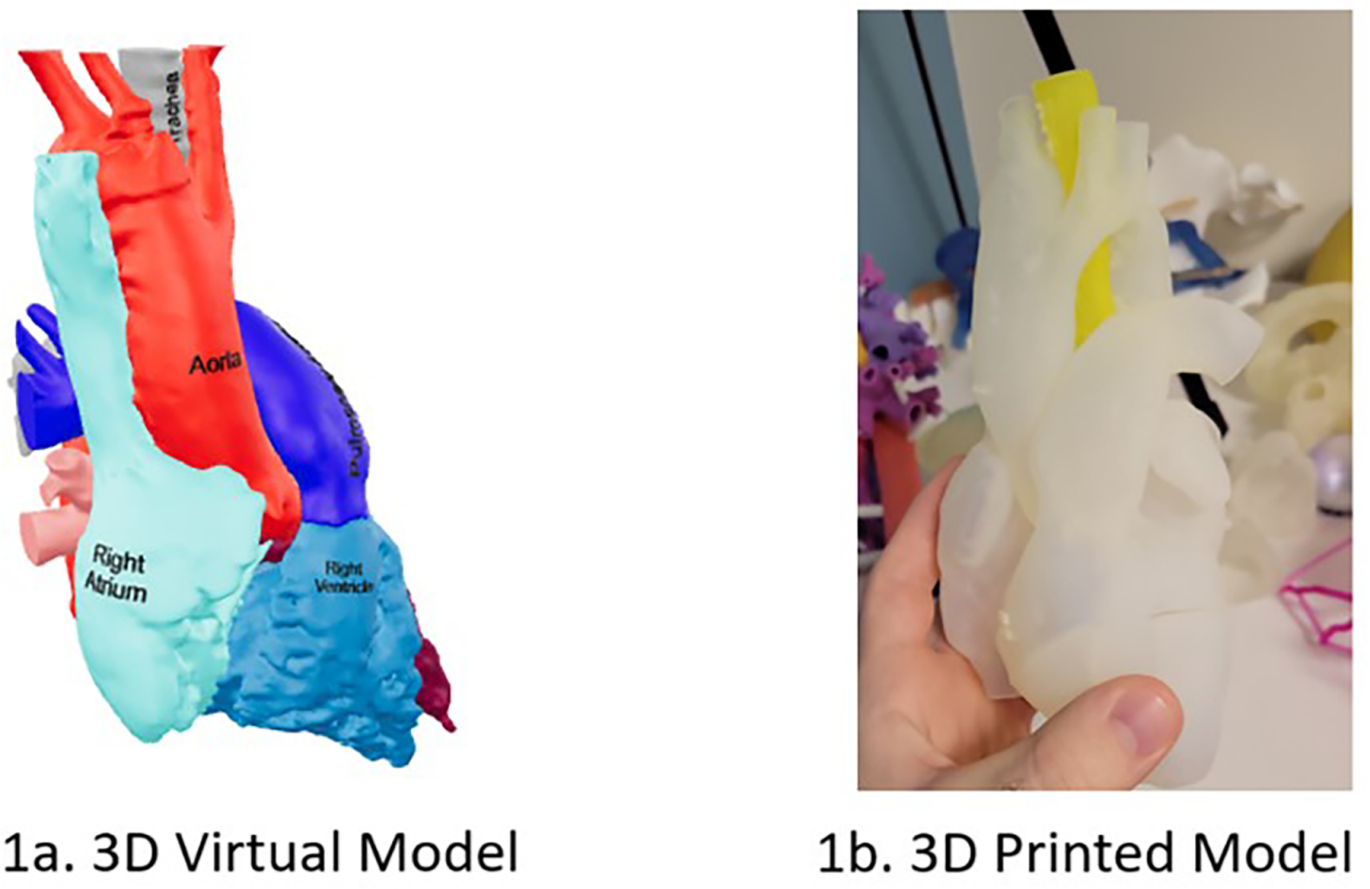
Patient cardiac diagnosis and demographic characteristics of test takers.

We created 3D printed and 3D virtual models (Figure 1) of various congenital heart defects (CHDs) representing patients seen in outpatient setting, including tetralogy of Fallot and coarctation of the aorta (Table 1). For each model, CT scan data was imported into Materialize Mimics software (version 25.0, Materialize, Leuven, Belgium). A threshold function was used to create the initial 3D model from the heart's blood pool. The heart model was segmented using this blood pool, allowing for the coloring of different components. The segmented model was then imported into Materialize 3-Matic, where we performed global and local smoothing operations. We labeled the structures in 3D based on the model's defect and performed a hollowing function for 3D printing to better visualize internal heart defects such as ventricular septal defects (VSDs) or an overriding aorta. The finished models were printed on a Stratasys J750 using Agilus 30 plastic. The same file used to create the 3D printed model was also used to make the 3D virtual model. Instead of hollowing the model, we exported the file to Blender, allowing us to color each cardiac segment and upload the file as a QR code that subjects could access by scanning with their smartphone.

### Study cohort and design

This study was approved by the Institutional Review Board. Family members of patients with CHD, as well as patients aged 15 years or older, were recruited. Most subjects were recruited from an outpatient pediatric cardiology clinic, with a few recruited from a neonatal intensive care unit. Subjects' demographic information was recorded, including age, race, ethnicity, gender, and education level.

Subjects were shown a normal echocardiogram and an echocardiogram image of a heart corresponding to the patient's cardiac defect. They completed a questionnaire to assess their knowledge of the cardiac defect, normal cardiac anatomy, and perceived understanding of their family member's CHD. First, subjects were asked to select the 2D diagram of the heart that represented the cardiac defect from four hearts with different CHDs. Second, they were asked to identify the right ventricle, aorta, left atrium, and tricuspid valve. Subjects then rated their understanding of normal heart anatomy and their understanding of the patient's heart condition on a five-point scale from “poor” to “excellent.” Finally, subjects rated the helpfulness of the model in aiding their understanding of the cardiac defect on a five-point scale from “not at all helpful” to “extremely helpful”.

Subjects were then randomly assigned to one of two groups: 3D printed model or 3D virtual model. The 3D printed model group received education using a 3D printed model of a normal heart and a 3D printed model representing the patient's cardiac defect. The 3D virtual model group received education using a 3D virtual model of a normal heart and a 3D virtual model representing the patient's cardiac defect. After the education session with the 3D model, subjects completed the same questionnaire. Additionally, subjects were asked to compare the helpfulness of the 3D model to the 2D image and respond to two questions about how likely they would be to use the 3D model to explain the cardiac defect to friends/family and to other medical professionals (e.g., emergency department physicians). One researcher (LZ) conducted all education sessions.

### Statistical analysis

The Cochran-Mantel-Haenzel test statistic based on rank scores, and controlling for education, was used to compare score/rating between the virtual and 3D printed groups. Additionally, the Wilcoxon signed-rank test was used to test for change within each group before and after introduction to the respective 3D model. Logistic regression fitted by the GEE method was utilized to account for correlation of response of test takers representing the same patient.

## Results

109 subjects participated in the study over a 2-month period, representing 73 individual patients (Table 1). Patients were only allowed to participate in the study if they were 15 years or older, therefore most subjects were the patient's parents (72.5%). Fifty-four subjects were randomized to receive education using a 3D printed model and 55 were randomized to receive education using a 3D virtual model. We found no initial significant differences between the two groups except for education level. Subjects that were randomized to receive education using a 3D virtual model had significantly higher levels of education. We controlled for this difference during analysis.

### Knowledge of CHD and normal anatomy

We analyzed subjects' performance on the anatomy portion of the questionnaire, comparing the subjects' knowledge after viewing the 2D image (Table 2). In analyzing, we found no statistically significant difference between the 3D printed and 3D virtual model groups. After viewing the 2D image, most subjects (*n* = 91, 83%) were able to identify the cardiac diagnosis when asked to select the one heart that corresponded with their CHD out of four hearts, and there was no significant increase in subject's ability to identify their cardiac diagnosis after viewing either 3D printed or 3D virtual model (*p* = 0.23, *n* = 47, 87% and *p* = 0.71, *n* = 49, 89% respectively) (Table 3). The subject's anatomy knowledge was assessed by asking them four questions to identify structures of normal heart anatomy. Subjects who initially obtained a perfect anatomy knowledge score (*n* = 44) were excluded from this portion of the analysis. Subjects in both 3D printed and 3D virtual model groups had a significant increase in their anatomy knowledge score between the first questionnaire (after the 2D image) and second questionnaire (after the 3D model) (*p* = 0.022 and *p* = 0.012 respectively) (Table 3).

**Table 2:** Post-2D questionnaire.

Variables	Printed 3D model	Virtual 3D model	Virtual vs. printed p-value*
Anatomy questions score, count (%)			*p* = 0.34
0–1	13 (24)	8 (15)	
2	11 (20)	10 (18)	
3	12 (22)	11 (20)	
4	18 (33)	26 (47)	
Understanding normal cardiac anatomy			*p* = 0.47
1 (poor)	1 (2)	2 (4)	
2	6 (11)	2 (4)	
3	22 (41)	22 (40)	
4	16 (30)	21 (38)	
5	9 (17)	8 (15)	
Understanding of condition			*p* = 0.47
1 (poor)	0 (0)	1 (2)	
2	4 (7)	0 (0)	
3	14 (26)	14 (25)	
4	17 (31)	25 (45)	
5 (excellent)	19 (35)	15 (27)	
Helpfulness of imaging to understand condition			*p* = 0.38
2 (not so)	0 (0)	2 (4)	
3 (somewhat)	15 (28)	11 (20)	
4 (very)	23 (43)	32 (58)	
5 (extremely)	16 (30)	10 (18)	

There was no significant difference in all the pre-questionnaire ratings and the anatomy question pre-score between the test takers in the printed and virtual groups.

* *p*-value for comparison of score/rating between Virtual and Printed from Cochran-Mantel-Haenszel test statistic based on rank scores, controlling for education.

**Table 3:** Comparison of responses post 2D and post 3D models.

Variables	Printed 3D model	Virtual 3D model	Virtual vs. printed p-value*
Able to identify diagnosis**, % yes (95% CI)			Virtual/printed: OR (95% CI)
Post 2D	80% (66%, 89%)	88% (74%, 95%)	1.86 (0.53, 6.55) *p* = 0.34
Post 3D	87% (76%, 94%)	90% (77%, 96%)	1.29 (0.36, 4.60) *p* = 0.69
3D vs. 2D: OR (95% CI)	1.72 (0.71, 4.16) *p* = 0.23	1.20 (0.46, 3.12) *p* = 0.71	Group*(3D-2D): *p* = 0.84
Anatomy questions, score change, count (%)			
All test takers	(*n* = 54)	(*n* = 55)	*p* = 0.67
Decreased (−2, −1)	9 (17)	7 (13)	
No change	29 (54)	35 (64)	
+1	10 (19)	10 (18)	
+2, +3	6 (11)	3 (5)	
Test change in score (3D-2D)***	*p* = 0.036	*p* = 0.17	
Test takers with 2D score <4	(*n* = 36)	(*n* = 29)	*p* = 0.65
Decreased (−1)	8 (22)	3 (10)	
No change	12 (33)	13 (45)	
+1	10 (28)	10 (34)	
+2, +3	6 (17)	3 (10)	
Test change in score (3D-2D)	*p* = 0.022	*p* = 0.012	
Understanding normal cardiac anatomy, rating change			
All test takers	(*n* = 53)	(*n* = 55)	*p* = 0.58
Decreased (−2, −1)	6 (11)	1 (2)	
No change	30 (57)	39 (71)	
+1	12 (23)	14 (25)	
+2, +3	5 (9)	1 (2)	
Test change in rating (3D-2D):	*p* = 0.016	*p* < 0.001	
Test takers with 2D rating <5	(*n* = 44)	(*n* = 47)	*p* = 0.76
Decreased (−1)	5 (11)	0 (0)	
No change	22 (50)	32 (68)	
+1	12 (27)	14 (30)	
+2, +3	5 (11)	1 (2)	
Test change in rating (3D-2D)	*p* = 0.009	*p* < 0.001	
Understanding of condition, rating change			
All test takers	(*n* = 54)	(*n* = 55)	*p* = 0.81
Decreased (−2, −1)	4 (7)	2 (4)	
No change	39 (72)	42 (76)	
+1	7 (13)	11 (20)	
+2	4 (7)	0 (0)	
Test change in rating (3D-2D)	*p* = 0.093	*p* = 0.076	
Test takers with 2D rating <5	(*n* = 35)	(*n* = 40)	*p* = 0.63
Decreased (−1)	4 (11)	1 (3)	
No change	20 (57)	28 (70)	
+1	7 (20)	11 (28)	
+2	4 (11)	0 (0)	
Test change in rating (3D-2D)	*p* = 0.093	*p* = 0.006	
Helpfulness of imaging 2D vs. 3D, rating change			
All test takers	(*n* = 54)	(*n* = 55)	*p* = 0.73
Decreased (−1)	5 (9)	5 (9)	
No change	31 (57)	28 (51)	
+1	18 (23)	17 (31)	
+2	0 (0)	5 (9)	
Test change in rating (3D-2D)	*p* = 0.004	*p* < 0.001	
Test takers with 2D rating <5	(*n* = 38)	(*n* = 45)	*p* = 0.78
Decreased (−1)	0 (0)	2 (4)	
No change	20 (53)	21 (47)	
+1	18 (47)	17 (38)	
+2	0 (0)	5 (11)	
Test change in rating (3D-2D)	*p* < 0.001	*p* < 0.001	

* *p*-value for comparison of change in score/rating between Virtual and Printed is from Cochran-Mantel-Haenszel statistic based on rank scores, controlling for education

** For response to “Able to Identify Diagnosis”, statistics were from logistic regression fitted by the GEE method to account for correlation of response of test takers representing the same patient, with odds ratio (95% CI) for Virtual relative to Printed and *p*-value based on model that included education as covariate.

*** *p*-value for the test for change (3D-2D) within group is from Wilcoxon signed-rank test

### Preference for 3d model

Subjects were asked to rate their understanding of normal anatomy. Both the 3D printed and 3D virtual model groups reported significantly higher self-rated understanding of normal cardiac anatomy compared to their ratings after viewing the 2D image (*p* = 0.009 and *p* < 0.001, respectively) (Table 3).

Next, subjects were asked to rate their understanding of their own or their family member's cardiac condition. Those in the 3D virtual model group who initially rated their understanding as less than 5/5 after viewing the 2D image later reported a significantly increased understanding (*p* = 0.006). However, there was no significant difference in the 3D printed model group on this measure (Table 3).

Finally, subjects in both the 3D printed and 3D virtual model groups found the 3D models more helpful (70% and 84%, respectively) compared to the 2D image (Table 4). Most subjects in both groups selected “agree” or “strongly agree” when asked if they would use the 3D model to explain their cardiac diagnosis to friends and family (98.1% and 98.2%, respectively). Similarly, most subjects in both groups selected “agree” or “strongly agree” when asked if they would use the 3D model to explain their cardiac diagnosis to other medical professionals (96.3% and 98.2%, respectively).

**Table 4:** Post 3D model questionnaire.

Variables	Printed 3D model	Virtual 3D model	Virtual vs. printed*
Helpfulness of 3D model compared to echo/CT			*p* = 0.33
1 Less helpful	1 (2)	1 (2)	
2 Equally helpful	15 (28)	8 (14)	
3 More helpful	38 (70)	46 (84)	
Use 3D model to explain to friends/family			*p* = 0.57
3 (Neither agree nor disagree)	1 (2)	1 (2)	
4 (Agree)	25 (46)	18 (33)	
5 (Strongly agree)	28 (52)	36 (65)	
Use 3D model to explain to medical professionals			*p* = 0.64
3 (Neither agree nor disagree)	2 (4)	1 (2)	
4 (Agree)	24 (44)	21 (38)	
5 (Strongly agree)	28 (52)	33 (60)	

* *p*-value for comparison of rating between Virtual and Printed Models from Cochran-Mantel-Haenszel test statistic based on rank scores, controlling for education.

## Discussion

This study is the first to compare the effectiveness of 3D printed models vs. 3D virtual models of congenital heart disease (CHD) in enhancing patient and family understanding. Existing literature predominantly focuses on the educational benefits of 3D printed models (4–8). By incorporating 3D virtual models, our research demonstrates that both types of models significantly improve patient and family comprehension and self-reported helpfulness.

While 3D printing is notably more time-consuming and costly, 3D virtual models offer a quicker, customizable alternative. These virtual models can be tailored to an individual's CT scan and shared via QR codes, allowing patients and families to easily explain the diagnosis to friends, relatives, and medical professionals.

The 3D printer used in this study was a high-end commercial printer and required expertise and time, however, given the advances in the availability of this technology, it may be more viable for individuals to print heart models on more consumer-accessible desktop printers. Additionally, these models can be customized to the printer's preferred design. Various resins can be used to depict the models along with the addition of color to highlight areas of the defect.

Our findings corroborate previous research on the educational impact of 3D models (4–8). Participants in both 3D model groups exhibited significant improvements in their knowledge of cardiac anatomy after viewing the 3D models compared to the 2D images. Additionally, subjects reported that the 3D models were more effective in aiding their understanding of their own or their family member's condition.

Interestingly, this study does not replicate the findings of Awori and colleagues in their 2021 study in which they concluded that printed models were of greater benefit to parents of children with CHDs (17). However, this study only included subjective measures of parent's preferences for the models, and did not include tests to assess their objective understanding of the CHD. Additionally, they stratified their analysis to differentiate between parents who perceived themselves to be more comfortable with modern technology and found that those who were more comfortable with modern technology rated 3D virtual models more highly than printed models. In future work, replicating our study with an added component of perceived comfort with modern technology could provide more insight into patient and family preferences.

Each model has its strengths and weaknesses. Based on our experiences 3D printed models are easier to manipulate but more costly to produce while 3D virtual models are easier to access while being cost effective. Additionally, some CHDs can be easily portrayed with the use of one model, however, other CHDs have substantial variety within one condition, such as TAPVR. This would require multiple models to accurately portray one condition, something that can be more easily done in a virtual environment specifically tailored to an individual patient's condition. Advanced 3D virtual models can be developed to enable patients and families to virtually dissect the heart, providing a clearer visualization of intracardiac defects. This level of customization is not feasible with 3D printed models. Other ways to visualize CHDs, such as virtual reality, could be used in future studies for educating patients and family members (18, 19).

## Conclusions

Given the lack of statistically significant differences between the two 3D groups, we propose that 3D virtual models are a more sophisticated option for the reasons outlined above. Furthermore, both 3D printed and virtual models are superior to 2D images for patient education and can be routinely implemented in clinical practice. Future research should focus on developing more advanced 3D virtual models and evaluating their effectiveness in clinical settings for patient and family education.

## Limitations

This study has several limitations. Subjects completed the questionnaire after viewing the 2D image and again after viewing the 3D models. While score increases may reflect a learning effect, subjects were not informed of the correct answers between the two tests. Additionally, a single heart model was used to represent each condition, which may not accurately reflect each patient's specific CHD. Future studies could improve upon this by creating heart models from each patient's CT scan, similar to the approach used by Biglino et al. in their 2017 study (7), and the Sun et al. study (20).

The subjects were aware that the study was comparing 2D models to 3D models. It is possible that preference for the 3D models could be impacted by social desirability bias. The increase in subjects’ knowledge was not likely impacted by social desirability, although it is possible that preference for the 3D model over the 2D model could have been influenced. This could be mitigated by randomly assigning the 2-D and 3- D models in future studies.

Although the recent census indicates that over 90% of individuals have broadband internet access at home and 90% have at least one computer (21), there are a small number of patients and families who would not be able to access the 3D virtual model at their home residence. In other countries with more limited access to the internet and 3D printers, fewer patients and clinicians may be able to utilize 3D models for education.

## Data Availability

The raw data supporting the conclusions of this article will be made available by the authors, without undue reservation.
